# South Carolina Dental Association

**Published:** 1874-10

**Authors:** 


### SOUTH CAROLINA DENTAL ASSOCIATION.

The annual meeting of the South Carolina Dental Association, was held at the usual time.

The meetings were held at the rooms of Dr. W. S. Brown, the President of the Association. The opening session commenced on Tuesday evening, May 16th, the President in the chair.

The roll of officers and members was called, and the minutes of last meeting read. All of the first answered to their names, and some ten or twelve of the members. After some preliminary business, the President read the address usual at the meetings of the Association; welcoming the country members to the city, and congratulating all on the success of the society and the decided importance of the meetings in relation to the advancement of the practice of dentistry. The address was replete with sentiments of interest and encouragement to those present, and a gentle admonition to to the absentees to consider seriously the importance of their presence not only for their their own, but for the benefit of those who come under their professional care. After some further business, the meeting adjourned until Wednesday morning at 10 o'clock.

### WEDNESDAY.

The Association assembled. President Brown in the chair. Roll of officers called—minutes read—when Dr. C. C. Patrick read a paper on "The effects of climate on the teeth." This paper elicited considerable discussion on the position taken by Dr. P., who held that climate had no direct effect in causing decay of the teeth; while several members held opposite views. This document being the doctor's first effort as an essayist before the Association, was decidedly meritorious; meeting the approval of all present, not excepting those opposed to his doctrine.

The next paper was on "The extraction of roots," in which the author gave as his opinion the necessity for extraction, in relation to the health of the mouth, as well as the entire economy—holding that a mass of diseased roots was one of the sources of many of the ills affecting humanity, and stating cases in practice in proof of his position.

Essays were also read by Drs. T. F. Chupein and John. L. Thompson, which were discussed by several of the members.

The Charleston members of the Association having chartered the yacht *Eleanor*, and having invited their country cousins to an airing around the harbor, the Association adjourned, and a number of gentlemen embraced the opportunity to view the location of some of the occurrences that have made the harbor of the Queen City noted for all time.

EVENING SESSION

Was called to order by President Brown, and a paper "Hæmorrhage after extraction," was read by Dr. Boozer—the doctor stating cases in practice, and also methods of treating them. Dr. Rice stated that in additon to topical applications, he had successfully used creosote internally, and had done so for several years. His practice was commented on by several of the members; some thinking the stoppage was more an incidental circumstance than from the internal use of creosote, yet all felt a desire to try the agent thus administered. The Association adjourned to meet at 10 o'clock on Thursday.

THURSDAY MORNING.

After the usual opening business, Dr. Wright brought to the notice of the Association the fact that the profession, in their organized bodies, in several of the states, had taken action in behalf of Dr S. C. Barnum, the discoverer of the use of the "rubber dam" now so generally in use with the profession for preserving both the material and cavities dry in the operation of filling teeth. Dr. W. stated that donations had been appropriated by other associations as a reward to Dr. B. for his generosity in making a free gift of his discovery to the profession instead of securing a patent for the same. A paper was circulated, and a generous amount subscribed for Dr. Barnum.

A preamble and resolutions in respect to the death or Dr. R. S. Whaley, of Newberry, were read and adopted, and ordered to be engrossed in the minutes. A renewal of the discussion on the remedies used in hæmorrhage after extraction followed, eliciting some remarks, from Drs. J. B. and C. C. Patrick, who doubted the effect ascribed to creosote, yet were prepared to give it a fair trial. Dr. Chupein introduced a patient with an apparent case of necrosis of the alveolus, reference made by other members to corresponding cases in their practice.

Dr. Brown introduced a patient with a fissure in the palate organ caused by a blow when the patient was seven years old. The injury extended to the germ of the eye tooth, which was found in a partly undeveloped condition in the alveolus; the incisor was also removed. An obturator had been worn several years. Drs. J. B. Patrick and W. S. Brown held the clinic.

Dr. I. H. Alexander read a paper on the use of celluloid (a preparation of gun-cotton) a base for artificial teeth, as a substitute for the vulcanite. Dr. A. gave his experience and expressed himself in favor of it. The Association awarded him the credit of being the first to use steam in place of oil—the use of the oil rendering the working of the celluloid more doubtful.

Dr. J. B. Patrick read a paper on "Dental Malformations and Irregularities" illustrating his cases with models and drawings on the black board. His remarks were delivered in his usual fluent style, and were listened to with marked attention by all the Association. The appliances for artificially restoring the loss of the velum and adjacent parts were beautifully executed.

Dr. Teague presented a new preparation for taking impressions, in place of wax or plaster. He also offered an appliance for operative dentistry, both of which were referred to a committee.

A committee was appointed, consisting of Drs Wright, Chupein, Bissell, Rice and Norwood, to draft an act to be submitted to the next legislature to regulate the practice of dentistry. Adjourned.

Oct-3

### FRIDAY.

The Association assembled at 10 o'clock the President in the chair. The committee reported the draft of an act for the regulation of the practice of dentistry which was read and unanimously adopted.

Drs. J. B. Patrick and T. T. Moore were added to the committee. Dr. J. R. Solomons presented a patient with the rare dental mal-formation of twin teeth, central incisors, connecting in both crown and root—lateral incisors being present in the jaw. The teeth were after a free discussion of the case, extracted.

The following officers were chosen by acclamation, to serve the ensuing year:

Dr. Theodore F. Chupein, Charleston, President; G. F. S. Wright, Pomria, First Vice President; M. Bissell, Camden, Second Vice President; C. C. Patrick, Charleston, Corresponding Secretary; J. W. Norwood, Greenville, Recording Secretary; T. W. Bonheur, Cheraw, Treasurer.

The city of Columbia was the place selected for the next meeting.

The meeting was harmonious throughout all its proceedings, all the members expressing themselves delighted with the urbanity of our late president who has filled his office with credit to himself and to the benefit of the Association, both mentally and physically. The only regret expressed by the members was in reference to the lukewarmness of those country members who did not attend, and sympathy for those who could not, from indisposition of themselves or families, give the assembly the benefit of their presence.

There were a few new members added to the list, namely Drs. McDavid, of Greenville; Rice, of Darlington; E. Solomons, of Sumpter besides one or two others.

WE HAVE White's new Burring Engine in Stock. Price, $50.00 SPENCER, CROCKER & Co.

